# Effects of SCH-23390 in Combination with a Low Dose of 17**β**-Estradiol on Anxiety-Like Behavior in Ovariectomized Rats

**DOI:** 10.1155/2014/847821

**Published:** 2014-02-23

**Authors:** Julia Fedotova

**Affiliations:** Laboratory of Neuroendocrinology, I. P. Pavlov Institute of Physiology of the Russian Academy of Science, 6 Emb. Makarova, Saint Petersburg 199034, Russia

## Abstract

The aim of this study was to explore effects on anxiety-like behavior of D_1_ dopamine receptor agonist, SKF-38393, and of D_1_ dopamine receptor antagonist, SCH-23390, given alone or in combination with a low dose of 17**β**-estradiol (17**β**-E_2_) to ovariectomized (OVX) rats. Two weeks after surgery, OVX rats began 14 days of treatment with the vehicle, a low dose of 17**β**-E_2_ (5.0 **μ**g/rat, s.c.), SKF-38393 (0.1 mg/kg, i.p.), SCH-23390 (0.1 mg/kg, i.p.), SKF-38393 plus 17**β**-E_2_, or SCH-23390 plus 17**β**-E_2_. The animals were tested in the black and white model (BWM) and the open field test (OFT). SCH-23390 (0.1 mg/kg, i.p.) alone or in a combination with a low dose of 17**β**-E_2_ (5.0 **μ**g/rat, s.c.) resulted in anxiolytic-like effect in OVX rats in the BWM. Repeated treatment with SCH-23390 and 17**β**-E_2_ profoundly increased anxiolytic-like effect of single substances exerted per se. Coadministration of SCH-23390 with 17**β**-E_2_ increased frequency of rearing and grooming in OVX rats in OFT. SKF-38393 (0.1 mg/kg, i.p.) treatment failed to alter anxiety-like behavior in OVX rats in the BWM. The results of the present study suggest that 17**β**-E_2_ and SCH-23390 interact to exert anxiolytic-like action and that each of these drugs can potentiate effects of each other.

## 1. Introduction

Anxiety is an adaptive response which detects and prepares an individual against a real or a potential threat [1]. There is an extensive literature highlighting close relationship between anxiety-like behavior and the mesolimbic dopamine (DA) system [2, 3]. Several preclinical and clinical data suggest that DA, acting on D_1_-like receptors, is one of the most important neuromodulators of fear and anxiety [4]. D_1_-like receptors family is composed of two different receptor subtypes, the D_1_ and D_5_ receptors [1]. High numbers of D_1_ receptors are located within caudate putamen (CPu), nucleus accumbens (NAc), and substantia nigra pars reticulata (SNr) with a less dense distribution in the amygdaloid regions [5]. Evidences suggest that D_1_ receptors in the CPu, NAc, and SNr facilitate motivated behavior [6, 7], while those in the amygdala are more involved in affective behavior [8, 9].

It is well established that stress activates the DA system and increases extracellular DA in the nucleus accumbens septi and the medial prefrontal cortex, inducing anxiolytic-like behavioral effects [10–12]. Besides, dopaminergic mechanisms alter behavioral responses to naturally anxiogenic environmental stimuli [3, 13]. In addition, both the DA system and anxiety-like behavior are dramatically altered by early-life stressors [14, 15] and withdrawal from chronic drug exposure [16, 17]. There is also evidence that stress-induced increases in DA metabolism can be attenuated by antianxiety drugs, such as diazepam and ICS 205930 (tropisetron) [11, 18, 19]. Also, animal studies indicated anxiolytic-like effects of D_1_ receptor antagonists on anxiety-related behavior which are dependent on behavioral model of anxiety and route of treatment [20–22]. However, the neurobiological mechanisms governing these relationships have not been fully established yet.

On the other hand, the activity of the dopaminergic neurotransmitter system is sensitive to modulation by the ovarian steroids. Estrogen depletion induced by ovariectomy results in a marked decrease in dopaminergic cell density in the brain [23]; this effect can be reversed by estrogen administration in 14 days after ovariectomy. Estrogen administration in 14 days after ovariectomy was still able to change activity of dopaminergic neurons, whereas it lost this capability after longer period of estrogen depletion [23]. Thus, changes in the peripheral levels of estrogen are associated with those in the activity of DA system [24, 25]. Indeed, estrogen affects the concentration of dopamine in specific brain areas and induces rapid changes in the response of striatal neurons to D_1_ dopamine receptor agonists [3] possibly by producing uncoupling of the DA receptor-G protein complex [4]. Prolonged estrogen administration induces downregulation of dopamine activity [23, 26] and produces a dopamine receptor supersensitivity [15, 27, 28] that results in a release from the inhibitory action of these receptors and enhancement of stimulated dopamine release. Estrogen also increased density of the dopamine reuptake protein [29, 30]. However, the mechanisms through which DA and estrogen signaling crosstalk and the role played by the DA receptor positive neurons still remain unclear.

The current experiment was conducted to clarify the effects of D_1_ receptor agonist/antagonist on anxiety-like behavior in ovariectomized rats on the black and white model (BWM). The BWM has been useful to screen substances with anxiolytic or anxiogenic potency [31] and to detect changes on anxiety-like indicators related with hormonal oscillations in cycling, pregnant, and lactating rats. This experimental model of anxiety is based on the innate preference of rats to explore a novel environment against their natural avoidance of a brightly illuminated open field. Thus, investigating the effects of dopaminergic drugs in the novel specific behavioral paradigm related to anxiety may lead to a greater understanding of the utility of DA and estrogen interaction in the mechanisms of anxiety disorders.

The main objective of this research is to study effects of D_1_ receptor agonist SKF-38393 and D_1_ receptor antagonist SCH-23390 injected chronically for 14 days alone or in combination with 17*β*-estradiol (17*β*-E_2_) on anxiety-like behavior in adult OVX rats. In the study, OVX rats and OVX rats with administration of 17*β*-E_2_ were used.

## 2. Methods

### 2.1. Animals

A total number of 70 adult female rats of Wistar strain (purchased from Rappolovo, Saint Petersburg, Russia) weighing 180–200 g were used. For at least a week prior to the experiments, the rats were housed six in a cage under standard environmental conditions: constant temperature of 23 ± 1°C, 60% humidity, 12 h light/dark cycle (light on 8:00 a.m.), food, and water ad libitum. All experiments were carried out in accordance with the guide for care and use of laboratory animals published by the National Institute of Health (National Research Council, publication 85-23, revised in 1996) and the Animal Welfare Assurance Renewal for I. P. Pavlov Institute of Physiology. The rationale, design, and methods of this study have been approved by the Ethical Committee for Animal Research, I. P. Pavlov Institute of Physiology. Experiments were carried out in a soundproof and air-regulated experimental room, to which animals were habituated, at least 30 min before each test.

### 2.2. Surgery

Ovariectomy was performed through an abdominal ventral incision under ethylic ether anesthesia. The complete extraction of the ovaries was corroborated by visual inspection. After surgery, females were retrieved in a community cage with other rats. Efficiency of 17*β*-E_2_ administration to OVX rats was controlled by vaginal smears. The ovarian phase as diestrus in intact rats was also detected by vaginal smears using light microscopy based on the predominant cell type. OVX animals were allowed to have 2 weeks for postoperative recovery before administration of drugs. After two weeks, all OVX rats were randomly assigned to each of the experimental groups and subjected to treatment and behavioral testing.

### 2.3. Drugs and Treatment

D_1_ receptor agonist, SKF-38393 hydrochloride, purchased from Sigma (D-047, Sigma Chemical Co., USA) and D_1_ receptor antagonist, SCH-23390, purchased from Sigma (D-054, Sigma Chemical Co., USA) were dissolved in sterile saline (0.9%). The estrogen 17*β*-E_2_, purchased from Sigma (E-8875, Sigma Chemical Co., USA), was dissolved in sterile sesame oil. All solutions were freshly prepared before each experimental series. Rats received SKF-38393 in dose of 0.1 mg/kg, i.p., SCH-23390 in dose of 0.1 mg/kg, i.p., and 17*β*-E_2_ in dose of 5.0 *μ*kg/rat, s.c. All preparations were chronically injected for 14 days once daily. Drugs were administered in 2 weeks after postoperative period following ovariectomy. Saline was injected i.p. to control 1 (intact rats) with the same procedure. Control 2 (OVX rats) received oil solvent injection in the same volume. All animals were gently handled by experienced keepers from the facility each day for a week prior to experimental procedures. Any environmental or physical stress was avoided in order to habituate the rats to manipulation. Rats subjected to drug or saline administration received an injection volume of 0.1 mL. All behavioral experiments were carried out in 45 min after the last injection of drug.

### 2.4. Experimental Design

OVX rats were divided into seven groups of 10 animals in each for performance of behavioral tests. Animals used for black and white model (BWM) and open field test (OFT) were the same. The two control groups constituted of intact rats in diestrus phase treated with saline i.p. (control 1) and OVX rats treated with oil solvent s.c. (control 2). The five other experimental groups were of OVX rats treated with 17*β*-E_2_; OVX rats injected with SKF-38393; OVX females injected with SCH-23390; OVX rats treated with SKF-38393 in combination with 17*β*-E_2_ in the same dose which was given to OVX rats; and OVX females treated with SCH-23390 daily in combination with 17*β*-E_2_ in the same dose which was applied to OVX females.

In a preliminary experiment, the dose-effect relationship of chronic administration of SKF-38393 or SCH-23390 to the OVX rats compared with placebo was studied in the locomotor activity test [32]. This experiment was carried out to verify test suitability in our experimental plan. For this reason, we selected three doses of SKF-38393 (0.05, 0.1, and 0.2 mg/kg) and SCH-23390 (0.05, 0.1, 0.2 mg/kg) and found the 0.1 mg/kg dose for two drugs being fully inactive in changing the locomotor activity. This dose was selected for succeeding experiments. Furthermore, this dose was considered as the minimum behavioral doses for OVX rats in the locomotor activity test [32]. Thus, this dose of dopaminergic drugs was selected for the BWM and the OFT. Ovariectomy markedly decreases estrogen level and 17*β*-E_2_ receptor activity in the different structures of the brain [27, 28]. In this connection, low dose of 17*β*-E_2_ may play a trigger role in activation of 17*β*-E_2_ receptor activity at hypoestrogenic syndrome [28]. Thus, we used a low dose of 17*β*-E_2_ in our present study. The low dose of 17*β*-E_2_ (5.0 *μ*g/rat, s.c.) was chosen from the studies performed by Estrada-Camarena and coworkers [33, 34].

## 3. Behavioral Tests

### 3.1. Black and White Model

The dimensions of the BWM were 80 × 40 × 40 cm. The box was further divided into two equal chambers (40 × 40 × 40 cm) by a barrier possessing a doorway (10 × 10 cm) that allowed the rats to cross freely from one chamber to the other. The black compartment was not illuminated, whereas the white compartment was completely illuminated by a 40 W white light. A video camera was installed above the illuminated compartment to record the rat activity in the box. Two independent observers later measured the behavioral variables. On the testing day, the rats were brought to the experimental room at 18:00 h (initiating the dark phase) and left for 1 hour to acclimatize to the novel surroundings. The BWM was initiated at 19:00 h, 1 hour after initiating the dark phase. After this time, the rat was placed into the middle of the black compartment facing the doorway and the rat behavioral activity was measured for 5 min. The evaluated variables were latency to the first entry into the white compartment, time spent in the white compartment, and frequency of entries into the white compartment. After each test session, the BWM was carefully cleaned and deodorized with a cleaning solution (Vekton, Russia, composition is ammonia 0.5%, ethanol 15%, extran 10%, isopropyl alcohol 5%, citrus aromatizing 19%, and distilled water 50.5% as v.v%).

### 3.2. Open Field Test

To evaluate the effect of dopaminergic substances on spontaneous locomotor activity, grooming, and rearing, the rats were submitted to a 5 min period to the OFT. An opaque Plexiglas cage (44 × 33 cm) with walls 20 cm in height was used. The floor was divided into 12 squares (11 × 11 cm). A video camera was installed above the cage to record the activity of the rats. Two independent observers measured the behavioral variables. After each test session, the OFT was carefully cleaned and deodorized with a cleaning solution (Vekton, Russia, composition is ammonia 0.5%, ethanol 15%, extran 10%, isopropyl alcohol 5%, citrus aromatizing 19%, and distilled water 50.5% as v.v%).

### 3.3. Statistical Analysis

All data were analyzed using unvariate random design analysis of variance and the post hoc Dunnett's test for multiple comparisons. A *P* value of 0.05 or less was considered as indicative of a significant difference. Where not indicated, ANOVA revealed no significant level of variance.

## 4. Results

### 4.1. Black and White Model

**Figure 1:**
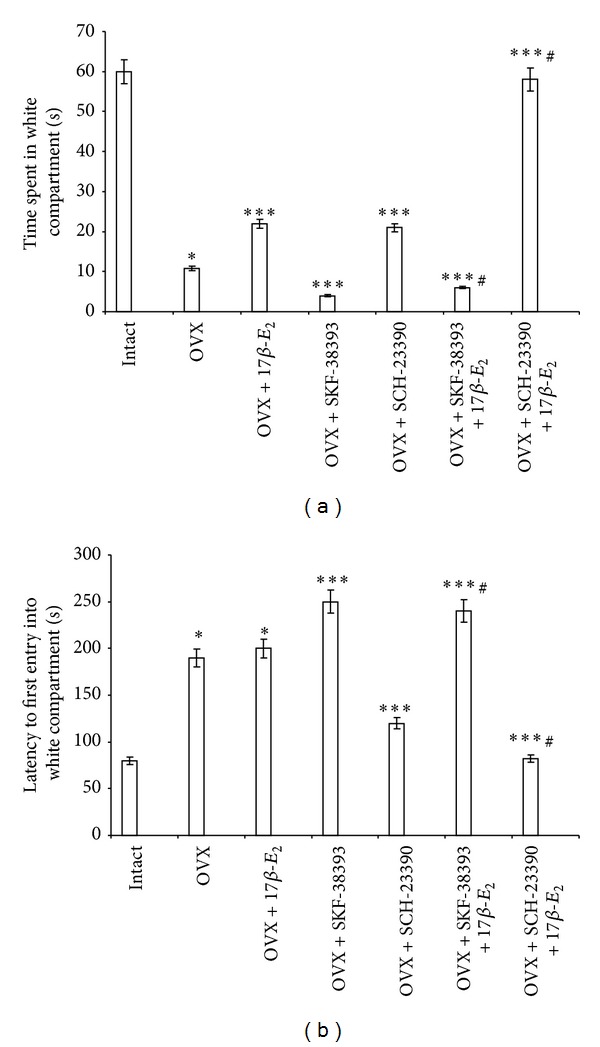
Effects of SKF-38393 (0.1 mg/kg, i.p.) and SCH-23390 (0.1 mg/kg, i.p.) on the anxiety-like behavior in OVX rats and OVX rats treated with low dose of 17*β*-E_2_ in the BWM. The time spent in white compartment (a) and the latency to first entry of experimental groups in the BWM. Columns represent the time spent in white compartment (a) as mean ± SEM (in sec) and latency to first entry into white compartment (b) as mean ± SEM (in sec). Each group was comprised of 10 rats. *P < 0.05 versus control intact rats (control 1); **P < 0.05 versus control OVX rats (control 2); ^#^ *P* < 0.05 versus OVX rats treated with 17*β*-E_2_.

The post hoc test revealed the differences among the groups for anxiety-like behavior in the BWM (*P* < 0.0001). The OVX rats treated by oil solvent only (control 2) demonstrated the markedly decreased time into the white compartment as compared to the control intact rats (control 1; *P* < 0.05; Figure 1(a)). The OVX rats given a low dose of 17*β*-E_2_ (5.0 *μ*g/kg) also spend more time into the white compartment as compared to the control groups 1 and 2, indicating that 17*β*-E_2_ in low dose is an anxiolytic agent (*P* < 0.05) (Figure 1(a)). Simultaneously, OVX rats treated with SCH-23390 (0.1 mg/kg) significantly spend more time in the white compartment as compared with the control groups 1 and 2 (*P* < 0.05, Figure 1(a)). Application of SCH-23390 in combination with a low dose of 17*β*-E_2_ in the OVX rats caused profoundly increase of the time into the white compartment (post hoc, versus control group 2 and OVX group treated with 17*β*-E_2_, *P* < 0.05). Thus, the combined administration of SCH-23390 with a low dose of 17*β*-E_2_ to the OVX females enhanced the positive effects of each preparation on the anxiolytic-like behavior in the BWM.

In contrast, the D_1_ receptor agonist SKF-38393 alone or SKF-38393 in a combination with a low dose of 17*β*-E_2_ significantly reduced the time spent in the white compartment compared with the control rats (*P* < 0.05, Figure 1(a)). Interestingly, OVX rats given SKF-38393 together with 17*β*-E_2_ demonstrated complete inhibition of the positive effect induced by 17*β*-E_2_ on anxiety-like behavior (post hoc, versus OVX group treated with 17*β*-E_2_, *P* < 0.05).

The post hoc test revealed differences among the groups for anxiety-like behavior in the BWM (*P* < 0.05). The OVX rats treated by oil solvent only (control 2) demonstrated the markedly increased first entry into the white compartment as compared to the control intact rats (control 1; *P* < 0.05; Figure 1(b)). The OVX rats given low dose of 17*β*-E_2_ (5.0 *μ*g/kg) failed to modify the latency to the first entry into the white compartment as compared to control OVX rats (*P* > 0.05, Figure 1(b)). The OVX rats treated with SCH-23390 (0.1 mg/kg) showed a significant decrease in the latency to the first entry into the white compartment compared with the OVX rats (*P* < 0.05, Figure 1(b)). The OVX rats given SCH-23390 plus 17*β*-E_2_ also demonstrated more decrease in the latency to the first entry into the white compartment (post hoc, versus OVX group and OVX group treated with 17*β*-E_2_, *P* < 0.05). The post hoc test showed that in OVX rats treated with SKF-38393 alone or SKF-38393 combined with 17*β*-E_2_, there was a significant increase in the latency to the first entry into the white compartment (post hoc, versus control intact group, OVX group, and OVX group treated with 17*β*-E_2_, *P* < 0.05).

**Figure 2:**
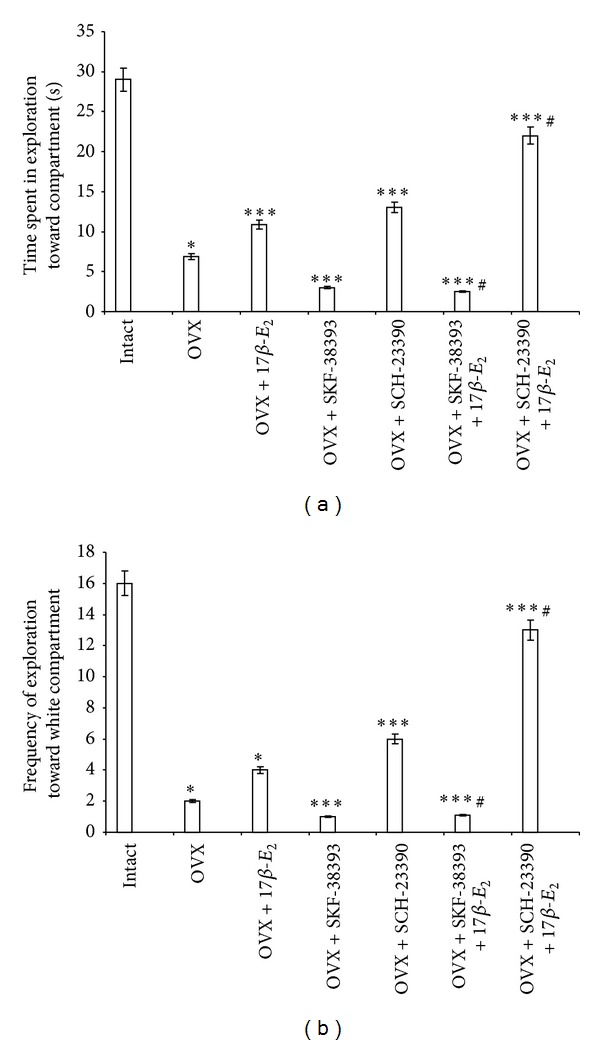
Effects of SKF-38393 (0.1 mg/kg, i.p.) and SCH-23390 (0.1 mg/kg, i.p.) on the anxiety-like behavior in OVX rats and OVX rats treated with low dose of 17*β*-E_2_ in the BWM. The time spent in exploration towards white compartment (a) and the frequency of explorations towards white compartment (b) of experimental groups in the BWM. Columns represent the time spent in exploration towards white compartment (a) as mean ± SEM (in sec) and the frequency of explorations towards white compartment (b) as mean ± SEM. Each group was comprised of 10 rats. *P < 0.05 versus control intact rats (control 1); **P < 0.05 versus control OVX rats (control 2); ^#^ *P* < 0.05 versus OVX rats treated with 17*β*-E_2_.

The post hoc test revealed differences among the groups for this parameter in the BWM (*P* < 0.05). The time spent in exploration towards the white compartment in the control OVX rats (control 2) was markedly decreased as compared to the control intact rats (control 1, Figure 2(a)). The OVX rats treated with 17*β*-E_2_ (5.0 *μ*g/kg) demonstrated a significant increase in the time spent in exploration towards the white compartment compared with the control OVX rats (*P* < 0.05), as compared to the control 2 (*P* < 0.05), but values of this parameter was significantly decreased as compared to the intact control group (Figure 2(a)). The post hoc test has revealed that OVX rats treated with SCH-23390 spent more time in exploration towards the white compartment as compared with the OVX rats (*P* < 0.05, Figure 2(a)). SCH-23390 in combination with 17*β*-E_2_ administered to the OVX rats resulted in a profound increase in the time spent in exploration towards the white compartment (post hoc, versus control OVX group and OVX group treated with 17*β*-E_2_, *P* < 0.05). Thus, the coadministration of SCH-23390 with a low dose of 17*β*-E_2_ to the OVX females improved effects of each preparation on anxiolytic-like behavior in the BWM. However, OVX rats treated with SKF-38393 alone or SKF-38393 in combination with 17*β*-E_2_ exhibited a significant decrease in the time of exploration towards the white compartment compared with the OVX rats (*P* < 0.05, Figure 2(a)). Interestingly, SKF-38393 administered with low dose of 17*β*-E_2_ in OVX rats completely blocked the positive effect of 17*β*-E_2_ on this parameter (post hoc, versus OVX group treated with 17*β*-E_2_, *P* < 0.05).

Similarly, the post hoc test revealed differences among the groups for this parameter in the BWM (*P* < 0.05). The frequency of explorations towards the white compartment in the control OVX rats (control 2) was markedly decreased as compared to the control intact rats (control 1, Figure 2(b)). The frequency of explorations towards the white compartment in OVX rats treated with a low dose of 17*β*-E_2_ (5.0 *μ*g/kg) was significantly increased as compared to the control 2 (*P* < 0.05), but values of this parameter was significantly decreased as compared to the intact control group (Figure 2(b)). The OVX rats treated with SCH-23390 displayed a significant increase in the frequency of explorations towards the white compartment compared with the control OVX rats (*P* < 0.05, Figure 2(b)). The coadministration of SCH-23390 and 17*β*-E_2_ resulted in a profound increase of the frequency of explorations towards the white compartment (post hoc, versus control OVX group and OVX group treated with 17*β*-E_2_, *P* < 0.05). Thus, coadministration of both preparations enhanced positive effects of SCH-23390 and low dose of 17*β*-E_2_ on anxiety-like behavior in the OVX females in the BWM. In contrast, the OVX rats treated with SKF-38393 alone or SKF-38393 plus low dose of 17*β*-E_2_ spent less time of exploration towards the white compartment compared to the control intact and OVX rats (*P* < 0.05, Figure 2(b)). Interestingly, coadministration of SKF-38393 and 17*β*-E_2_ to the OVX rats completely blocked the positive effect of 17*β*-E_2_ on this parameter (post hoc, versus OVX group treated with 17*β*-E_2_, *P* < 0.05).

### 4.2. Open Field Test

**Figure 3:**
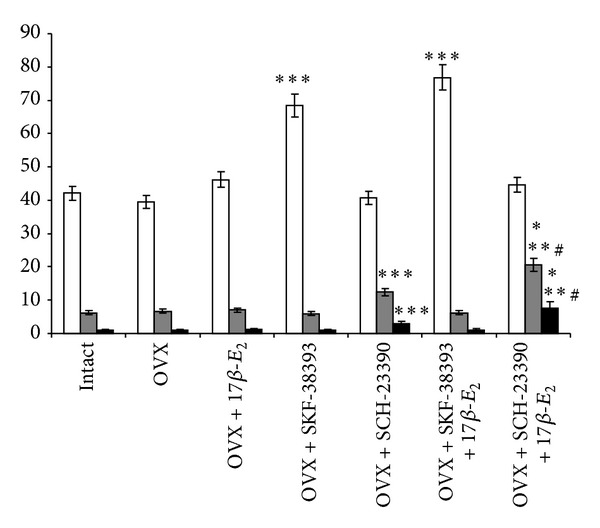
Effects of SKF-38393 (0.1 mg/kg, i.p.) and SCH-23390 (0.1 mg/kg, i.p.) on the behavior in OVX rats and OVX rats treated with low dose of 17*β*-E_2_ (5.0 *μ*g/rat, s.c.) in the open field test for 5 min. Columns represent the behavioral parameters as mean ± SEM. Light columns represent crossing, grey columns represent rearing, and dark columns represent grooming. Each group was comprised of 10 rats. *P < 0.05 versus control intact rats (control 1); **P < 0.05 versus control OVX rats (control 2); ^#^ *P* < 0.05 versus OVX rats treated with 17*β*-E_2_.

The post hoc test revealed differences among the groups for behavior in the OFT (*P* < 0.05). The ovariectomy or application of 17*β*-E_2_ in a low dose (5.0 *μ*g/kg) failed to modify behavioral reactions of OVX rats as compared to the intact control females in the OFT (*P* > 0.05, Figure 3). Neither SCH-23390 alone nor SCH-23390 plus 17*β*-E_2_ led to change of crossing behavior in the OVX rats (post hoc, versus control group, OVX group, and OVX group treated with 17*β*-E_2_, *P* > 0.05). However, the post hoc test revealed that OVX rats treated with SCH-23390 demonstrated a significant increase in the frequency of rearing and grooming as compared to the control intact and OVX rats (*P* < 0.05, Figure 3). Coadministration of SCH-23390 with 17*β*-E_2_ resulted in higher frequency of rearing and grooming in the OVX rats (post hoc, versus control OVX group and OVX group treated with 17*β*-E_2_, *P* < 0.05, Figure 3). On the contrary, administration of the D_1_-receptor agonist, SKF-38393 alone, or SKF-38393 with low dose of 17*β*-E_2_ significantly enhanced crossing behavior (post hoc, versus control intact group, control OVX group, and OVX group treated with 17*β*-E_2_, *P* < 0.05, Figure 3). We found that Neither SKF-38393 nor SKF-38393 plus 17*β*-E_2_ led to altered rearing and grooming behavior in OVX rats in the OFT (post hoc, versus control intact group, control OVX group, and OVX group treated with 17*β*-E_2_, *P* > 0.05, Figure 3).

## 5. Discussion

The main result of the present study was that SCH-23390 significantly decreased anxiety-related behavior in OVX rats treated with low dose of 17*β*-E_2_, suggesting that anxiolytic-like effect of SCH-23390 in the BWM is independent of the presence of 17*β*-E_2_. However, it should be emphasized that combination of SCH-23390 with a low dose of 17*β*-E_2_ is more effective for correction of anxiety-like behavior in the OVX rats. Also, the results of the present study demonstrated that a low dose of 17*β*-E_2_ was not effective on the latency to the first entry into the white compartment. One may assume that such dose of 17*β*-E_2_ is not enough for correction of this behavioral parameter. 17*β*-E_2_ has been reported to possess anxiolytic-like activity in animal models of anxiety, although the mechanism for such effect has not been fully established yet [27, 28]. Some studies have demonstrated that 17*β*-E_2_ exerted an anxiolytic-like effect preferentially through the modulation of dopaminergic receptors [35, 36].

Since we aimed to detect anxiolytic-like effect of SCH-23390 and 17*β*-E_2_ coadministration, we selected a low dose of each drug to emphasize the synergistic effect of both preparations [33, 34]. Our results showed that SCH-23390 (0.1 mg/kg, i.p.) exerted a significant anxiolytic-like effect in the BWM. Intriguingly, we observed a profound anxiolytic-like activity in OVX rats treated with SCH-23390 plus 17*β*-E_2_. To the best of our knowledge, there is no behavioral research for interaction of estrogen and SCH-23390 in anxiety-like behavior. The results of this study demonstrate that 17*β*-E_2_ and SCH-23390 interact to enhance anxiolytic-like efficacy.

Several lines of evidence suggest that DA is released in several brain regions such as the amygdala and the prefrontal cortex under stress conditions. By acting on D_1_- or D_2_-like receptors, DA is involved in physiological processes subserving affective behaviors and emotional learning [4]. Biochemical studies have indicated that some anxiolytic drugs (diazepam and tropisetron) might attenuate stress-induced increase in DA metabolism [11, 18, 19]. Also, animal studies indicated anxiolytic-like effect of D_1_ receptor antagonists, such as SCH-23390 [1, 37, 38]. Some data suggest that selective D_1_ antagonists have anxiolytic-like effect in the EPM in mice, while stimulation of D_1_ receptors prevents anxiolytic-like effect of D_1_ antagonists as well as other agents [39, 40]. Here, our present results are in good agreement with this data, showing that some dopaminergic drugs acting as DA receptor antagonists are also active as anxiolytics.

We did not find any dependence between effect of SKF-38393 or SCH-23390 on anxiety-like behavior and effect of these drugs on locomotor activity. Since neither 17*β*-E_2_ nor SCH-23390 significantly influenced behavior in locomotor activity test, the anxiolytic-like synergy observed in the BWM cannot be attributed to altered motor activity. Data of the BWM and the locomotor test considered together indicate that interaction between 17*β*-E_2_ and SCH-23390 is a mood effect rather than a motor effect. Thus, our results suggest that D_1_ receptor antagonist may possess anxiolytic-like activity as it was shown in some reports [39, 40].

Estrogen exerts a wide range of actions in the mammalian brain that extend far beyond its classical role as regulator of the hypothalamic-pituitary-gonadal axis [27, 28, 41]. These actions include neurotrophic effects, such as promotion of cell survival [42], modulation of synaptogenesis [28] and axonal and dendritic sprouting [23], and enhancement of neurogenesis [27, 28]. In addition, estrogen modulates certain brain functions by affecting neurotransmitter levels within distinct neuronal populations as well as the expression of receptors and second messengers [24, 25]. Indeed, estrogen affects the concentration of dopamine in specific brain areas and induces rapid changes in the response of striatal neurons to D_1_ and D_2_ dopamine receptor agonists [43], possibly by producing an uncoupling of the D_1_ receptor-G protein complex [44]. Prolonged estrogen administration induces downregulation of presynaptic dopamine activity [30, 45] and produces a dopamine receptor supersensitivity [45–48] that results in a release from the inhibitory action of these receptors and enhancement of stimulated dopamine release. Estrogen may, thus, increase activity of dopaminergic neurons through a combination of mechanisms, including a reduction in inhibitory control exerted by autoreceptors [45–48], an increase in activity of dopamine transporter [30], inhibition of Ca^2+^ influx [49], uncoupling of the D_1_ receptor-G protein complex [44], and modulation of neuronal plasticity [42, 50].

**Figure 4:**
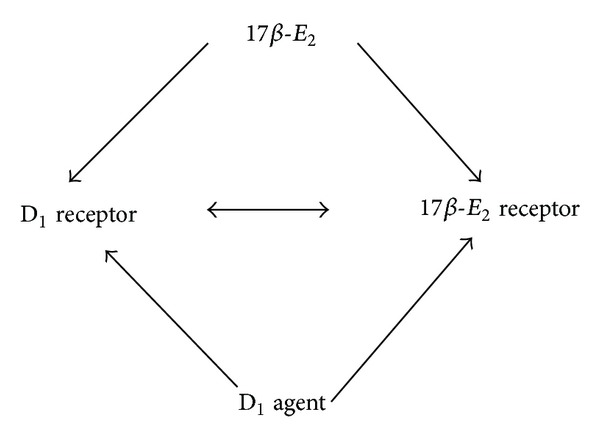
Possible mechanism of interaction between dopaminergic drug and 17*β*-E_2_.

Currently, we do not know how exactly estrogen and DA modulate anxiety-related behavior. However, it is tempting to assume that a direct or indirect receptor-receptor interaction, via intracellular signaling pathways, might be involved. Effects of SCH-23390 application could be considered as part of estrogen withdrawal syndrome or manifestation of its genomic action. It can be assumed that low dose of 17*β*-E_2_ may directly influence both 17*β*-E_2_ receptor and D_1_ receptor (Figure 4). The precise mechanism by which SCH-23390 alone or in a combination with 17*β*-E_2_ induced positive effect on anxiety-like behavior remains to be established. Nevertheless, the present data add new facet to crosstalk between neurotransmitter dopaminergic and hormonal estrogen systems, within the framework of anxiety-related behavior. Estradiol likely has a stimulatory effect on the activity of dopaminergic neuronal system, playing a permissive role in SCH-23390 effect. Although it is not possible to assign specific role to estrogen in regulation of dopaminergic activity, observation that dopaminergic neurons are sensitive to a synergistic action of circulating steroid level provides a clue to understanding how fluctuations in ovarian hormone levels may amplify or ameliorate the symptomatology of psychiatric disorders characterized by altered mood and emotional states as well as the hypoestrogenic differences in drug sensitivity [27, 28].

The main interesting result from this study is that SKF-38393, selective D_1_ receptor agonist, inhibited anxiolytic-like effect of 17*β*-E_2_ in BWM. Some evidence has shown that estrogen receptors (ERs) can be activated by dopaminergic ligands acting on D_1_ receptors [26, 41]. One may assume that SKF-38393 prevents binding of 17*β*-E_2_ with ERs resulting in blockade of 17*β*-E_2_ positive action on anxiety-like behavior in OVX rats (Figure 4). Here, diverging effects of SKF-38393 and SCH-23390 in OVX rats can be related to changed metabolism of DA, expression of D_1_ receptors, expression of 17*β*-E_2_ receptors, and to their binding ability in the brain structures directly related to mood functions. There are a number of alternative possibilities worth considering.

In summary, D_1_ receptor antagonist, SCH-23390, administered alone or in a combination with a low dose of 17*β*-E_2_ results in anxiolytic-like effect in OVX rats. Repeated treatment with SCH-23390 and low dose of 17*β*-E_2_ profoundly enhanced anxiolytic-like effect the single substances per se. D_1_ receptor agonist, SKF-38393, failed to modify anxiety-like behavior in OVX rats in BWM. In addition, SKF-38393 blocked anxiolytic-like effect of 17*β*-E_2_ in OVX rats. Further research is needed to elucidate the detailed mechanism by which SCH-23390 and 17*β*-E_2_ exert synergistic effect on anxiety-related behavior.
